# Accelerating research to keep pace with the rising incidence of coccidioidomycosis

**DOI:** 10.1128/msphere.00082-26

**Published:** 2026-09-25

**Authors:** Christina M. Homer

**Affiliations:** 1 Division of Infectious Diseases, Department of Medicine, Oregon Health & Science University 6684, Portland, Oregon, USA; 2 Department of Molecular Microbiology and Immunology, Oregon Health & Science University 6684, Portland, Oregon, USA; Shenzhen Institute of Synthetic Biology, Shenzhen, China

**Keywords:** mycology, coccidioidomycosis, fungal pathogenesis

## Abstract

The number of infections caused by the fungal pathogen *Coccidioides* is rising. In tandem, the number and breadth of researchers studying this important dimorphic fungal pathogen are growing as well. This review focuses on recent advances in the field and identifies areas primed for ongoing rapid growth, with the goal to improve treatments for coccidioidomycosis as soon as reasonably possible. These areas include building genetic tools, high-throughput drug screening, developing immunomodulatory therapies for coccidioidomycosis, and incorporating research methodologies that can be performed outside of the biosafety level 3 laboratory.

## INTRODUCTION

The overall number of fungal infections worldwide is rising sharply (1–3). There are many reasons: an increasing number of people living with immunosuppression (4), escalating antibiotic use (5), and changing geographic ranges in the setting of climate change (6), among other contributing factors. Coccidioidomycosis, the infection caused by two *Coccidioides* species (*immitis* and *posadasii*), is no exception (7–9). Cases of coccidioidomycosis have increased more than 7-fold in 25 years (10, 11). The latest data available at the national level in the United States is from 2023; in that year, 21,037 cases were reported to the CDC. However, a recent study estimates the actual incidence to be 10 to 18 times higher due to underdiagnosis, misdiagnosis, and underreporting (12). Increasing coccidioidomycosis incidence is theorized to be due to multiple factors: (i) the environment becoming more permissive to growth and dispersal of *Coccidioides* (13–15); (ii) increased geographical distribution due to climate change (6, 13); (iii) population growth in endemic areas leading to soil disturbance as houses are built on arid land (13, 16); and (iv) a larger at-risk population due to more use of immunosuppressive medications in medical care (13, 17–19) and a higher total population living in endemic areas (13). Additionally, it is plausible that increased testing rates and evolving case definitions (20) are contributing, although the limited data available suggest testing rates remain low among health care providers (21, 22).

**Fig 1:**
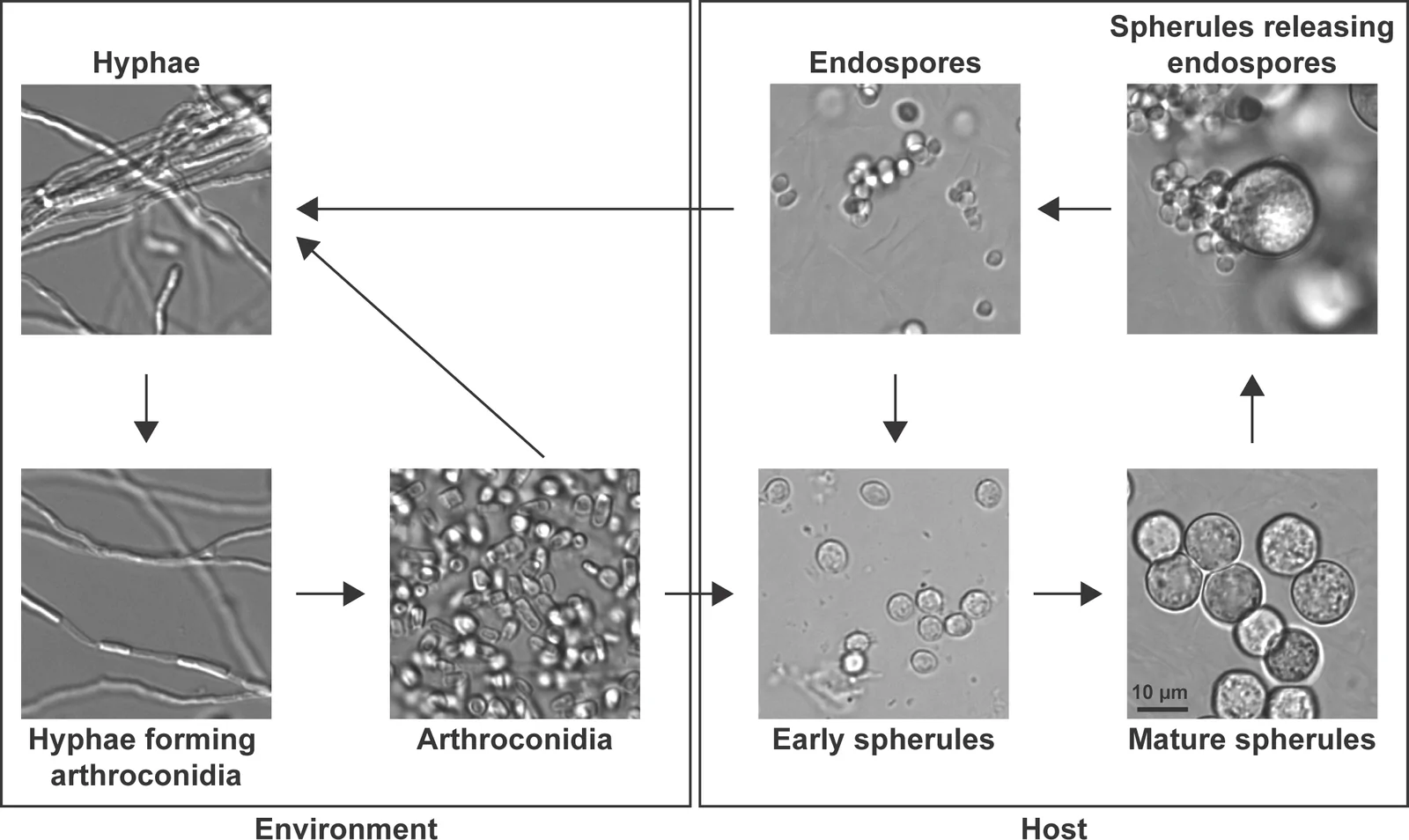
Micrographs of *Coccidioides* development. In the environment, *Coccidioides* grows as hyphae, which produce arthroconidia, asexual infectious spores. Under environmental conditions, arthroconidia can germinate into hyphae. When arthroconidia are inhaled by mammalian hosts, they germinate, round, and grow into early spherules. As spherules mature, they increase in size and internally segment to form asexual endospores. Spherules release endospores, and each endospore can develop into another spherule or germinate into hyphae. All images are at the same scale.

*C. immitis* and *C. posadasii* are found in arid regions of the Americas (6). These fungi grow in the soil as hyphae and produce asexual spores, called arthroconidia, which are the infectious particles that can be aerosolized and inhaled by mammals (23). Once in the lung of a mammalian host, arthroconidia germinate into the host morphology of *Coccidioides*, the spherule (Fig. 1). Arthroconidia round and enlarge as they become early spherules, then continue to grow, undergo nuclear replication, and internally segment. Once mature, a spherule is filled with endospores, asexual spores unique to the host lifecycle (23). Mature spherules rupture and release endospores, each of which can go on to form another spherule.

Based on epidemiologic surveys, about 60% of exposures to *Coccidioides* lead to subclinical or asymptomatic infection in humans (24). About 40% of those exposed develop pulmonary infection, and 0.5–3% (25, 26) are estimated to develop disseminated infection, a manifestation of disease in which *Coccidioides* can involve almost any organ. Disseminated infections cause 20–40% mortality despite current treatments (27–29). Currently, there are only two classes of antifungals approved for treatment of coccidioidomycosis: azoles and polyenes (30). Azoles are the first-line treatment recommended for most *Coccidioides* infections, including meningitis (30). However, azoles appear to suppress rather than clear the infection, even after years of treatment (31), necessitating lifelong therapy. Among polyenes, Amphotericin B is the only agent currently available to treat systemic fungal infections, including coccidioidomycosis. Due to high toxicity, its use is typically reserved for azole treatment failure, severe and rapidly progressive disseminated infections, or when azoles are contraindicated in the first trimester of pregnancy (30). Amphotericin B can only be administered through intravenous and intrathecal routes (32), limiting its use outside of hospitals and specialized clinics. Despite these limitations, intrathecal amphotericin B has been successfully used to cure *Coccidioides* meningitis in some cases (32).

While *Coccidioides* was discovered in 1892 (33), research that could underpin diagnostic modalities and novel treatments has been hindered by multiple hurdles. The classification of *Coccidioides* as a select agent (34) and the need to work with wild-type strains under biosafety level 3 (BSL-3) containment (35) have made it difficult for many labs to perform research on this fungus. Technical hurdles include low-throughput genetic editing (36) and non-standard microbial growth conditions (37–40). Since no other organism is known to grow as a spherule, our current understanding of spherule biology is limited, even though spherules are fundamental to *Coccidioides* virulence. *Coccidioides* spherule-specific proteins often have no clear orthologs in other fungi (41) since these proteins likely evolved to play a specific role in the spherule. Therefore, dissecting their function is paramount but involves the laborious task of characterizing proteins of unknown function without clues from orthologs in better-studied fungi.

A recent influx of funding for *Coccidioides* research has spurred new directions aimed at detecting, treating, and preventing coccidioidomycosis. As the field grows, researchers are building toolboxes for inquiry into this pathogen with a pragmatic approach and designing studies to interrogate key aspects of fungal biology that will improve how coccidioidomycosis is managed clinically. This review focuses on the recent advances leading to an increased pace of research in the *Coccidioides* field and identifies areas primed for ongoing rapid development.

## INCREASING EFFICIENCY OF GENETIC APPROACHES—BUILDING A GENETIC TOOLBOX

As with other organisms (42–44), the advance of the CRISPR/Cas gene editing technology has been an opportunity for increasing the throughput of genetic experiments in *Coccidioides*. Historically, genome editing in *Coccidioides* was accomplished through homologous recombination with long (1 kb) homology arms (36) or through *Agrobacterium tumefaciens*-mediated genome editing (45). While these methods generated critical mutants for the field, they are laborious and inefficient at generating the desired mutant. As gene editing is undertaken in multinucleate morphologies of *Coccidioides* (23), multiple rounds of passaging transformants under selection are necessary to obtain homokaryons. This further increases the amount of work when transformations are inefficient, and many transformants need to be screened to obtain the desired mutant. Additionally, *Agrobacterium*-based transformations frequently lead to ectopic integrants (46–48).

A transformation protocol using CRISPR/Cas9 paired with a DNA repair template using 35–50 bp microhomology arms was adapted from *Aspergillus fumigatus* for use in *Coccidioides* (49). Recently, the first five CRISPR/Cas9-generated *Coccidioides* mutants were published, using 50–100 bp homology arms (50). Adoption of various CRISPR/Cas systems has allowed researchers to create mutant strains more easily in a wide range of organisms, including nonhuman primates (51), mice (52, 53), plants (54–56), insects (44), and fungi (43, 57–59). Among fungi, CRISPR/Cas systems have enabled increased efficiency and precise control of genome editing in medically and industrially important *Aspergillus* species (60), the fungal pathogen *Histoplasma (61, 62),* and model mushrooms *Schizophyllum commune* and *Coprinopsis cinerea* (59). CRISPR/Cas systems have enabled gene disruption and editing for the first time in non-laboratory strains of *Aspergillus* (63) and commercially valuable mushroom species (64–66). In the pathogenic yeast *Cryptococcus neoformans*, CRISPR/Cas9 use increased efficiency of genome editing (nearing 100%) despite use of shorter homology arms (67–69). Given the significant increase in mutant generation through CRISPR/Cas use in other organisms (and non-model fungi in particular), this will likely be the beginning of many more *Coccidioides* mutants being generated.

Increasing flexibility in genome engineering should lead to the development of a genetic toolbox for studying the biology of this fascinating fungus. Preliminary data suggest very few off-target editing events, and, with further optimization, the number of passages to obtain a homokaryon may be able to be decreased or potentially eliminated (Homer, unpublished results). In addition to generating individual deletion mutants to interrogate gene function, this same protocol can be adapted to tag proteins with epitopes or fluorescent proteins. Such tags would obviate the need to generate custom antibodies for individual proteins, an expensive and time-consuming undertaking, and enable a wide range of experiments from subcellular localization and western blotting to co-immunoprecipitation and immunoprecipitation–mass spectrometry. Strains expressing fluorescent proteins could be employed to determine spore fate (analogous to the *Aspergillus fumigatus* FLARE strain [70]) and track interactions with host cells during infection (71–73). Fluorescent reporters specific to hyphae and spherules would enable higher-throughput plate-based screens designed to reveal the genes required for the *Coccidioides* dimorphic switch. Fluorescent reporter strains could be used to detect host-specific gene expression (74) or sense host cell conditions, such as the reporter strain that was used to interrogate *in vivo* zinc levels during *Histoplasma* infection (75).

Finally, increasingly efficient genetic manipulations are an opportunity to generate additional strains that can be safely manipulated under BSL-2 containment and increase the number of researchers able to contribute to *Coccidioides* research. The *C. posadasii* C735 ∆*cts2*/∆*ard1*/∆*cts3* (TKO strain) (76) and the *cps1*∆ vaccine strain (48) have been widely used in BSL-2 labs. However, these mutants are unable to complete spherulation, limiting their use in research questions focused on spherule biology and fungal pathogenesis. A strain derived from *C. posadasii* (known as the S/E strain) was recently reported to grow exclusively as spherules and endospores in Converse media but unexpectedly transitioned to hyphal growth upon inoculation in tissue culture media designed to mimic host conditions (77). While the authors were approved to work with this strain in BSL-2 containment at their institution, the strain retained virulence in an insect model of infection. Therefore, further work to understand whether this strain retains virulence in a mammalian model of infection is necessary before it can be widely used in other BSL-2 labs. CRISPR/Cas9 can be leveraged to create additional mutants that could be safely manipulated in BSL-2 containment but preserve spherule biology, including generation of auxotrophic mutants in *Coccidioides*. Auxotrophic strains have been powerful tools in the study of other pathogens that normally require BSL-3 containment (*Mycobacterium tuberculosis* [78–80] and *Histoplasma* [62, 81, 82]). While careful validation would be required, pyrimidine and purine auxotrophs in *Coccidioides* would likely be avirulent due to nutrient limitations during mammalian infections but grow as wild type *in vitro* when requisite nutrients are supplemented.

## DESIGNING HIGHER-THROUGHPUT SCREENS TO IDENTIFY NEW ANTIFUNGAL TREATMENTS

There has been exciting and significant progress in the field to develop a vaccine for coccidioidomycosis. This progress on vaccine development has been reviewed in detail elsewhere (83, 84) and will not be the focus of this review. However, the changing geography of the endemic region (6) and sporadic infections that occur outside of the endemic region (85) will make it challenging to offer vaccination to all individuals at risk. Additionally, people living with immune compromise are more susceptible to disseminated coccidioidomycosis and also less likely to develop protective immunity after vaccination (86). Therefore, if a vaccine becomes available, there will be an ongoing need to treat breakthrough *Coccidioides* infections. Reasons for treatment failure are not fully understood (28), including how the unique spherule morphology of *Coccidioides* may contribute. Further uncertainty stems from clinical antifungal susceptibility testing being performed on hyphae rather than spherules (87). Current antifungal classes used to treat *Coccidioides* (polyenes and azoles) (30) target ergosterol, a component of the plasma membrane of fungi (88, 89). While ergosterol has been detected in the plasma membrane of *Coccidioides* hyphae (90), the chemical constituents of the plasma membrane of the spherule remain uncharacterized. Azoles are fungicidal for other endemic mycoses, but they do not effectively eliminate *Coccidioides* (31). Therefore, it is not clear if the currently used antifungals are optimally targeting the spherule; new antifungal treatments effective against this unique host morphology are needed.

*De novo* drug design requires significant resource investment, and most candidate compounds do not ultimately become approved drugs (91). There has not been an effort to develop a dedicated antifungal targeting *Coccidioides* spherules specifically, likely due to the geographically limited nature of coccidioidomycosis and high biocontainment capabilities required to work safely with *Coccidioides*. Novel antifungals developed as broad-spectrum antimycotics are being tested against *Coccidioides*, and some of these agents have shown recent promise. Olorofim, the first antifungal to target dihydroorotate dehydrogenase, recently demonstrated effectiveness in treating coccidioidomycosis in a Phase 2b clinical trial (92–94). A newly designed polyene, EL219 (95), demonstrates decreased toxicity while maintaining broad antifungal activity in both *in vitro* and *in vivo* studies, including in an intracranial murine model of coccidioidomycosis (96). Fosmanogepix, a first-in-class broad-spectrum antifungal drug targeting the cell wall enzyme Gwt1, has also been found to have activity against *Coccidioides in vitro* and *in vivo* (97), with promising results published in a small case series involving 11 patients with coccidioidomycosis (98). However, geographic restriction and outcome frameworks that do not accurately capture the slow response to *Coccidioides* treatment have made it difficult to design clinical trials (99) and secure funding to test these agents in patients, as reviewed in depth recently (100).

Instead, the advantages of drug repurposing are being leveraged, testing U.S. Food and Drug Administration (FDA)-approved compounds for activity against new targets (101). This approach was first used to evaluate a library of 1,280 FDA-approved drugs in combination with the first-line antifungal fluconazole (102) against hyphae from the attenuated TKO strain. This study design, where novel compounds are added to standard treatment, sets the stage for an “add-on design” clinical trial. “Add-on” trials have several attractive qualities: (i) they facilitate higher recruitment since patients do not have to avoid standard therapies, a useful characteristic for trials involving a geographically restricted infection; and (ii) the “add-on” design inherently selects for compounds with mechanisms of action distinct from current therapies, which is useful for discovering synergistic treatments or overcoming resistance to current therapies (103). Importantly, authors tested several top screen hits in a murine coccidioidomycosis model using wild-type *Coccidioides*. Despite screening TKO mutant hyphae, two of the three combinatorial treatment hits reduced the pulmonary fungal burden of a wild-type strain. A similar screen performed on spherules may increase the number of hits and diversify the types of compounds that can be repurposed to treat coccidioidomycosis even further.

In fact, such an approach was used in two subsequent studies. First, a group used the wild-type *C. posadasii* strain C735 to develop 96-well plate assays and screened 7,722 FDA-approved drugs for inhibition of spherule development (104). The authors chose early synchronous spherules, hypothesizing inhibition at this early stage of development would likely block further pathogenesis. Growth and cell viability in the presence of the tested compounds were measured using XTT, a colorimetric indicator of metabolic activity, since standard OD600 growth curves are challenging given spherules’ propensity to settle and clump. This screen revealed 27 potential drugs with activity against *Coccidioides*. Twelve hits were further characterized based on favorable IC50 values. To investigate the mechanism of action for individual drugs, the authors developed a flow cytometry assay to characterize fungal cell changes in the presence of drugs using dyes to evaluate the cell wall, nucleic acids, and mitochondria. The team then screened the same chemical library against the attenuated TKO strain and showed a strong correlation between the response of wild-type and the TKO mutant to most compounds. In a subsequent study, researchers scaled up screening capacity using 384 well plates and the TKO mutant, laying the foundation for a truly high-throughput screening in *Coccidioides*. However, unlike the study discussed above, hits from this screen were not tested in an animal model, and some top hits are not sufficiently absorbed to treat systemic disease.

While these approaches were used for drug screening, similar methods could be leveraged to ask fundamental questions about *Coccidioides* biology. High-throughput screens using XTT could be performed on wild type and a collection of mutant strains treated with a vast array of chemical compounds, not only FDA-approved drugs. This chemical genomics approach has been successful in identifying the function of uncharacterized genes in other fungi (105, 106). Fluorescent dyes and antibodies specific to cellular pathways or structures could be used with flow cytometry as a readout in screens of mutant collections as well. Using CRISPR/Cas9-generated mutants, these approaches should provide functional insights for the many unannotated *Coccidioides* genes.

The top drug candidates identified by these two groups did not overlap, likely due to differences in screening approaches, indicating the potential to uncover a broad array of additional compounds active against *Coccidioides*. The challenge then becomes higher-throughput validation strategies. Murine models are time and resource intensive. If a screen results in many promising hits, most labs need to prioritize the top one to five hits for validation in mice. The recent establishment of *Galleria mellonella* as a low-cost, fast model for coccidioidomycosis drug susceptibility testing (107) could help further prioritize hits. Arthroconidia injected into *Galleria* moth larvae developed into spherules and endospores, although authors also observed some filaments that may be hyphae. Larvae treated with established antifungals survived longer than untreated controls during *Coccidioides* infection. Authors then tested two drug candidates in this model and found one candidate was toxic to the larvae, but the other candidate conferred a survival benefit. Further work is needed to determine whether drug activity in this insect model of coccidioidomycosis translates to a murine model and, ultimately, to humans. If so, this would be a cheap and faster model to initially screen candidate compounds before entering pre-clinical and clinical drug development pipelines.

## MODULATING THE HOST IMMUNE RESPONSE TO IMPROVE COCCIDIOIDOMYCOSIS OUTCOMES

While drug development attempts to more effectively target the pathogen, modulating the host immune response to *Coccidioides* also shows promise in improving infection outcomes. An initial case report demonstrated that off-label FDA-approved treatment with interferon-gamma and an anti-IL-4-receptor-antibody (dupilumab) was able to resolve pathologic Th2 skewing to treat severe disseminated coccidioidomycosis (108). Now, a case series of 204 patients with coccidioidomycosis demonstrates that this pathologic Th2 skewing is more common than previously recognized, affecting 23% of patients with disseminated coccidioidomycosis in their study (109). Surprisingly, this effect was most pronounced in male patients with disseminated coccidioidomycosis, indicating a patient population that may benefit from functional immunological testing and treatment to suppress Th2 skewing.

The PD-1 immune checkpoint was also identified as a potential host therapeutic target in two studies using single-cell RNA-seq of host cells in *Coccidioides*-infected mouse lungs. Despite using different fungal strains and different timepoints (500 wild-type arthroconidia at days 5, 9, and 14 post-infection [110] versus 10^6^ mutant TKO arthroconidia at 24 h post-infection [111]), both studies identified a robust population of neutrophils expressing high levels of the transcript encoding PD-L1. PD-L1 has been targeted in multiple cancers by immune checkpoint inhibitors that block its association with the checkpoint protein PD-1, increasing the immune system’s ability to control or clear cancer cells (112). Immune checkpoint inhibitors have been proposed as treatment for fungal infections (113), and, intriguingly, an abstract was recently published indicating a modest survival benefit from anti-PD-L1 treatment in a murine model of coccidioidomycosis (114). Using orthogonal immunophenotyping, a different group also found that infection by wild-type *Coccidioides* induced recruitment of PD-L1-expressing granulocytes (115). Their study employed functional assays and adoptive transfers to determine that PD-L1-expressing cells were detrimental during coccidioidomycosis, increasing their promise as treatment targets. Finally, a study comparing the single-cell transcriptomic response of the human airway epithelium to wild-type *Coccidioides posadasii* or *Aspergillus fumigatus* revealed that *Coccidioides* stimulation resulted in less potent expression of neutrophil chemoattractants than *Aspergillus* (116), raising the question of whether immunomodulators targeting increased neutrophil recruitment and increased neutrophil activity could be used to treat coccidioidomycosis. As the FDA has approved agents to target PD-1/PD-L1 and modulate neutrophil recruitment, these promising findings warrant further investigation and could be translated quickly to clinical trials.

## SCIENCE OUTSIDE OF THE BSL-3 LABORATORY

While extremely important for studying a BSL-3 pathogen, work inside of the BSL-3 laboratory inherently limits some experimental approaches. For important safety considerations, there are restrictions on use of sharps and aerosolizing procedures under BSL-3 containment. Additionally, dedicated equipment needs to be purchased for the BSL-3 laboratory that cannot be reused for BSL-1 or BSL-2 experiments, increasing the cost of research. Finally, necessary safety procedures within the BSL-3 add time and can make protocols with very early or very long timepoints impractical to perform safely. Therefore, experiments that can safely be done outside of the BSL-3 laboratory provide opportunities to use more sophisticated experimental approaches and accelerate discovery.

Historically, one common approach to study individual *Coccidioides* proteins has been to produce recombinant proteins in *Escherichia coli* (117–122). This often yields high levels of protein for downstream structural and biochemical studies. However, the *E. coli* prokaryotic expression platform does not always produce active *Coccidioides* enzymes, so eukaryotic expression systems have been occasionally used (123–125). Additionally, native *Coccidioides* proteins have been isolated from fungal cultures, although this approach does not fully bypass the need for a BSL-3 facility (117, 121, 126). All these approaches are fairly low-throughput ones and require individual optimization for each protein target being expressed. Recently, a group has published the use of a nucleic acid programmable protein array (NAPPA) for high-throughput expression of *Coccidioides* proteins using an *in vitro* transcription and translation system (127). The NAPPA allowed researchers to express 708 *Coccidioides* proteins simultaneously and screen serum samples from *Coccidioides*-infected and uninfected dogs against them. The screen identified 221 fungal antigens, including a novel seroreactive antigen. While NAPPA technology has been used to study antibody reactivity and spur vaccine development, it can also be used to query protein–protein interactions (128) or read out enzymatic activity if colorimetric or fluorescent probes are available (129). Applying this technology to further questions in *Coccidioides* pathogenesis, such as identifying additional antigens in human patients, probing host–pathogen protein–protein interactions at scale, or identifying fungal proteins with a specific enzymatic activity, will advance the field substantially.

For conserved *Coccidioides* proteins, heterologous expression systems can also be employed outside of the BSL-3 laboratory. This has proven to be a powerful method of determining protein function in other fungi (130–134). Heterologous expression of *Coccidioides* genes in multiple eukaryotic hosts has been successfully used to determine the genes encoding ornithine decarboxylase and orotate phosphoribosyl transferase (135–137). Given these successes, this powerful tool should be applied more broadly. Using the *Saccharomyces cerevisiae* (138) genome-wide gene deletion collection*,* conserved *Coccidioides* proteins could be systematically expressed in *S. cerevisiae* mutants lacking the predicted ortholog. Heterologous expression mutants can then be evaluated to determine whether they restore that ortholog’s function.

In plant fungal pathogens, heterologous expression of fungal avirulence (Avr) proteins in plant hosts has been a very successful strategy to identify the paired plant resistance (R) gene (139). An R protein recognizes a specific Avr protein and triggers a protective plant immune response. Identification of these protein pairs has been done one-by-one until recently, when a high-throughput method was developed. Researchers expressed pools of Avr candidates from *Puccinia graminis*, the fungus that causes wheat stem rust, in seven wheat protoplast lines expressing different R genes (140). In total, the group screened 696 candidate Avr genes against seven R genes and found four Avr-R gene interactions, including two novel Avr-R gene pairs and the two previously known Avr-R gene pairs for *P. graminis*. This >50% hit rate for R genes screened is a significantly higher yield than that of previous approaches. Expanding on this concept of pooled heterologous expression screens, a recent study expressed a large collection of bacterial, viral, and parasitic effectors in a human cell line and discovered multiple pathogen effectors that manipulate host cell pathways during infection (141). Although it remains to be determined how much of the *Coccidioides* lifecycle is completed inside host cells, a recent study demonstrated that macrophage uptake is a key signal promoting *Coccidioides* spherule formation, suggesting a critical intracellular phase of spherule development in the host (142). Therefore, heterologous expression of candidate spherule effectors in host cells may identify host pathways targeted by the fungus and identify novel treatment targets.

Finally, bioinformatic approaches using published data do not require access to BSL-3 laboratories. Given the complexity of modern sequencing data sets, whole-genome sequencing or transcriptomics studies are published with focused analyses. There are many other analyses possible on the same data demonstrated in a recent publication that discovered “islands” of spherule-expressed genes physically clustered in the *Coccidioides* genome and signatures of natural selection acting on spherule-specific genes, suggesting interesting candidate virulence factors for further study (143). Given the rising pace of “omics” publications in *Coccidioides* experiments, this approach will become increasingly high-yield and can be used to prioritize directions for future research, an approach discussed in detail in a recent review (144).

## CONCLUSIONS

The pace of *Coccidioides* research continues to accelerate as the field grows, spurred by increasing interest in studying the fungus responsible for rising cases of coccidioidomycosis. Leveraging recently published advances in genetic editing, newly developed high-throughput and large-data experimental techniques in *Coccidioides* and other fields, and BSL-3-independent approaches, future research will begin to translate foundational science into changes in clinical practice for patients living with coccidioidomycosis.
